# In vitro development of embryos from experimentally Kerack-addicted Mice

**Published:** 2017-07

**Authors:** Elham Mohammadzadeh, Fatemeh-Sadat Amjadi, Mansoureh Movahedin, Zahra Zandieh, Zohreh Nazmara, Neda Eslahi, Peymaneh Shirinbayan, Hamid Reza Asgari, Nahid Azad, Maryam Salimi, Morteza Koruji

**Affiliations:** 1 *Shefa Neuroscience Research Center, Khatam Alanbia Hospital, Tehran, Iran.*; 2 *Department of Reproductive Biology and Anatomical Sciences, Faculty of Medicine, Shahid Beheshti University of Medical Sciences, Tehran, Iran.*; 3 *Cellular and Molecular Research Center, Iran University of Medical Sciences, Tehran, Iran.*; 4 *Department of Anatomical Sciences, School of Medicine, Iran University of Medical Sciences, Tehran, Iran. *; 5 *Anatomical Sciences Department, Medical Sciences Faculty, Tarbiat Modares University, Tehran, Iran. *; 6 *Pediatric Neuro-Rehabilitation Research Center, the University of Social Welfare and Rehabilitation Sciences, Tehran, Iran.*

**Keywords:** Addiction, Preimplantation, Embryo development, Apoptosis, Mouse

## Abstract

**Background:**

Prenatal drug exposure, as a common public health concern, is associated with an increased risk of adverse effects on early embryo development.

**Objective:**

To investigate the *in vitro* development of - embryo from experimentally Kerack-addicted mice.

**Materials and Methods:**

Twenty-five female mice were studied in five groups: control, vehicle, and three experimental groups of Kerack-dependent mice (I, II, and III) which received different doses of Kerack for 14 days. After the establishment of addiction model (7 days), experimental groups I, II, and III were given Kerack intraperitoneally at the doses of 5, 35, and 70 mg/kg, twice a day for a period of 7 days, respectively. The vehicle group received normal saline and lemon juice whilst the control group just received water and food. Morulae were obtained through oviduct flashing. The survived embryos were cultured in T6+ 5mg/ml bovine serum albumin. The developmental rates up to hatched stage daily and embryo quality (differential staining and Tunnel staining) were also assessed

**Results:**

The developmental potential of embryos obtained from the addicted mother was significantly decreased in comparison with control group. There was a significant reduction in the rate of blastocyst formation in the high dose Kerack dependent group. However, in addicted mice there was reduction in the total cell number (40.92% vs. 65.08% in control) and, inner cell mass percentage (17.17% vs. 26.15% in control) while apoptotic cells numbers were increased (7.17 vs. 1.46 in control) (p<0.05).

**Conclusion:**

The Kerack addiction during pregnancy retards preimplantation development and induces apoptosis.

## Introduction

Substance abuse is an important problem worldwide affecting the mothers and the growing infants (1, 2). The teratogenic role of illicit substance drugs is known during human development; these drugs include marijuana, alcohol, tobacco, opiates, and cocaine. Currently, 25% of deaths are related to alcohol, tobacco, or illicit drug use (3). Pregnant women who use illicit substances have high frequencies of fetal morbidity (disease) and mortality (death) (4, 5). Based on some national data sources, the number of drug abusers in Iran is 1.2-2 million people that they are generally young and in reproductive age with a mean age of 33 yr old (6-9).

The second most commonly used substance is Kerack which is a street name for a newer type of opiates (a purer form of heroin) whose usage is ever-growing (10). Kerack is different from crack cocaine in other countries (11). Kerack analysis by different studies shows that its components consist of acetaminophen, acetylcodeine, caffeine, codeine, heroin, morphine, papaverin, tebain and also some impurity (11, 12). It can be smoked, inhaled or injected by the users (13). The discontinuation of Kerack can produce withdrawal symptoms more quickly in the dependent persons, thereby requiring more frequent injection (14).

So far, the number of studies on the effects of drugs on the male reproductive system is more than the female reproductive system. In our previous study, we examined the effects of Kerack on sperm parameters and structures and also we investigated genes involved in CatSper ion canals. The results showed that Kerack has devastating effects on sperm as a male gamete- in the process of fertility (15). There are few studies about the effect of drugs on the embryo and female reproductive system. These studies mainly focused on some kinds of drugs such as cocaine and marijuana, and fewer studies were conducted on other drugs. 

Khoradmehr and colleagues evaluated effects of prenatal methamphetamine (MA) administration during gestational days on mice (2). They showed that MA abuse during pregnancy can cause morphological and histological changes in mice fetus but the exact mechanism remains unclear. Another study on pregnant women showed that women who consume cocaine/crack during pregnancy, have lower incomes and are more likely to use alcohol. During pregnancy, fetal weight, height, in addition to newborn baby skull size can be decreased in these cases. Thus fetal exposure to cocaine/crack in early pregnancy can reduce fetal symmetrical growth (16). 

Kaufmann and Armant examined the effects of cocaine on the fetus in vitro and two-cell embryos were exposed to cocaine in culture environment. They concluded that cocaine could influence on the embryonic development in blastocyst stage and that high doses of cocaine inhibit the growth of one- and two-cell embryos (17). It seems that the assessment of embryo preimplantation in drug abusers is necessary and is relevant to fertility potential, preservation of fertility, and preimplantation embryo quality. 

Regarding the major differences between these illicit drugs, the growing number of drug abusers, and the effects of the drug on the male reproductive system (18). This study attempted to investigate the effect of Kerack on the early embryo development and quality, employing addicted mice model.

## Materials and methods

**Animals**

This experimental study was performed in Iran University of Medical Sciences (IUMS) between 2014 and 2015. After obtaining NMRI mice (male and female) from Razi Vaccine and Serum Research Institute (Karaj, Iran), they were acclimatized to the laboratory conditions for two weeks in the animal house of IUMS before the experiments started. 

**Embryos**

Embryos were collected from six to eight-weeks-old female NMRI mice. Mice were superovulated with an intraperitoneal injection of 7.5 IU pregnant mare's serum gonadotropin (PMSG; hypra, Spain), followed by 7.5 IU human chorionic gonadotropin (HCG; 6000IU, Bioniche, Australia), given 48 hr apart. After the second injection, females were mated with males from the same strain and were inspected for the presence of vaginal plug the following day. Mice with the presence of vaginal plug considered as pregnant and sacrificed 78-82 hr post- human chorionic gonadotropin (hCG) injection by cervical dislocation. Morulae were flushed from oviduct and uterus horns using T6 medium supplemented with 5 mg/ml bovine serum albumin (BSA; Sigma, USA). Morphologically normal embryos were washed and pooled in the medium before use.

**Preparation of addicted animal and testing of the withdrawal syndrome**

To investigate the effect of Kerack on preimplantation embryos, we provided an addicted model to study drug abuse similar to patients who consume Kerack as described previously (15). So, all mice in experimental groups (I, II, III) were addicted to Kerack for 7 days. The addiction procedure and dose selection were performed as previously described (15). For the first 3 days, they respectively received intra-peritoneal (IP) Kerack with doses of 20, 25 and 30 mg/kg of their body weight. On the 4^th^/5^th^, 6^th^, and 7^th^ day, they received Kerack at the 40, 60 and 80 mg/kg (IP) dosage twice a day, respectively. 

The withdrawal syndromes were measured by injection of Naloxone. In addition, the addicted mice were tested for previously identified behavioral characteristics of the mice opiate abstinence syndrome such as jumping, shaking, and exploring by injection of Naloxone HCl (5 mg/kg) 2 hr after the first administration of Kerack on the seventh day (19). Five addicted mice were randomly selected and checked for withdrawal syndrome just for one time.

**Experimental design**

**Figure 1:**
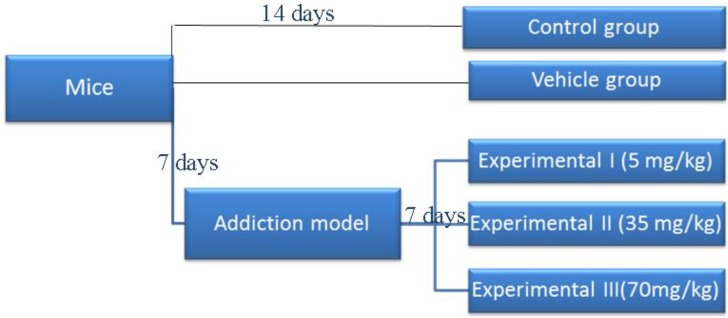
Schematic experimental design

The animals were weighed and randomly divided into five groups (Figure 1): (i) Control group did not receive any drug; (ii) Vehicle group received only 220 µl of normal saline and lemon juice as a solvent for Kerack (2.6 µl/ml, applied as drug solvent); (iii). Experimental group I received Kerack at a dose of 5 mg/kg after addiction to Kerack; (iv) Experimental group II received Kerack at a dose of 35 mg/kg after addiction to Kerack and (v) Experimental group III received Kerack at a dose of 70 mg/kg after addiction to Kerack (IP), for the remaining 7 days, twice a day. 

After 14 days of treatment with Kerack, mice were superovulated, mated and sacrificed by cervical dislocation for embryos collection as mentioned before. Embryos obtained from all groups were allocated into the drops of T6 medium with 5 mg/ml BSA. Afterward, the experiments were replicated five times for embryos (n=10-15 embryo/drop) while being observed and recorded every 24 hr for 4 days.

**Determination of Inner cell mass number in embryos**

To determine the blastocyst cell numbers from each group, embryos were placed in drops supplemented with 500 µl of propidium iodide 100 µg/ml (Sigma, Germany) at 37^o^C for 20-50 sec. There were approximately 10 embryos per group that was in the blastocyst stage 48 hr after cultivation. This was followed by incubation in 500 µl of bisbenzimide 25 µg/ml (Hoechst 33342, Sigma, Germany) in absolute ethanol, overnight at 4^o^C. Embryos were mounted on microscope slides with glycerol, a coverslip was placed on the top of the embryos, and they were initially examined to evaluate the number of cells. 

Under fluorescence microscopy (excitation filter at 420 nm, barrier filter at 365 nm), the outer trophectoderm cells were identified by the pink fluorescence of propidium iodide, whereas the Inner Cell Mass (ICM) were recognized by the blue fluorescence of bisbenzimide. The numbers of ICM and trophectoderm cells nuclei were counted under an inverted fluorescence microscope (IX71, Olympus, Japan).

**TUNEL staining to determine apoptotic cell number**

TUNEL assay was carried out using an In Situ Cell Death Detection Kit (Roche, Mannheim, Germany) according to provided protocol. Briefly, following less washing in Phosphate-buffered saline (PBS) (Sigma-Aldrich, USA), the blastocysts were fixed in 4% paraformaldehyde solution (Wako, Japan) and treated with 0.1% Triton ×100 solution (Sigma, Germany) for 3 min. Then, the blastocysts were primarily incubated in TUNEL solution (Roche, Germany) at 37^o^C for an hour according to the manufacturer's instructions. 

Negative control embryos were incubated only in fluorescent solution without enzyme to ensure the absence of labeling. For the positive control, a number of blastocysts prior to incubation with TUNEL staining solution were incubated with 50 μg/ml DNase I solution (Sigma, Germany) for one hour and then treated with TUNEL solution. Finally, all blastocysts were counterstained with 4; 6-diamidino-2-phenylindole (DAPI) in PBS (Santa Cruz Biotechnology) diluted 1:2 for 10 sec. Then, samples were evaluated under a fluorescence microscope, and the number of TUNEL-positive cells in embryo was calculated and photographed using a fluorescent microscope at ×40 magnification. Finally, the percentages of apoptotic cells were calculated.

**Ethical consideration**

All animal experimentation protocols were approved by IUMS Animal Care and Use Committee (Code: IR.IUMS.rec.1390.12452).

**Statistical analysis**

Statistical comparison of developmental rates in this experimental study was performed using Chi-square analysis between five groups utilizing SPSS software, version 18.0 (Statistical Package for the Social Sciences, version 18.0, SPSS Inc, Chicago, Illinois, USA). Mean±SD of numbers of blastomer, ICM, and apoptotic cells were analyzed by one-way analysis of variances (ANOVA) and Tukey’s test. Results were assumed significant at p≤0.05.

## Results

**Establishment of addicted model in mice via intraperitoneal injection**

The model used happened to be an addicted model in mice via intraperitoneal injection. The Kerack administrated mice showed euphoria signs after substance injection including tail stiffness and twirling around the cage over 20 min. Withdrawal signs (jumping, shaking, exploring, and also scratching and hand licking) were increased in naloxone-administered mice. Therefore, the addicted model in mice via intraperitoneal injection was established.

**The effect of Kerack on development of morulae in culture following 96 hr**

During the cultivation period, consumption of Kerack decreased the development of morulae embryos obtained from addicted mothers (Table I). The comparison of control and vehicle groups indicated that the Kerack solvent (lemon juice) did not have any noticeable side effects on the developmental stages of preimplantation during the pregnancy and addiction periods. At the first 24 hr in addicted groups, the ceased embryos at the morula stage were significantly more than the control group (p≤0.001). The delay was compensated at subsequent times during the cultivation.

The comparison between the addicted and control groups showed that 24 hr after culturing in all three experimental groups, no embryos hatched. Besides, cessation in the morula stage and degenerated embryos was significantly more in all three experimental groups compared to the control group. Although in 48, 72, and 96 hr the development of embryos increased, the hatched embryos were noticeably less and the degenerated embryos were strongly more than the control group (p=0.028). Totally, the results showed that consuming Kerack in addicted mothers could lead to the death of embryos in the culture and cause a significant decrease in the rate of hatched embryos which is an important factor for the implantation.

**The effect of Kerack on blastomere, ICM, and apoptotic cells number**

**Figure 2:**
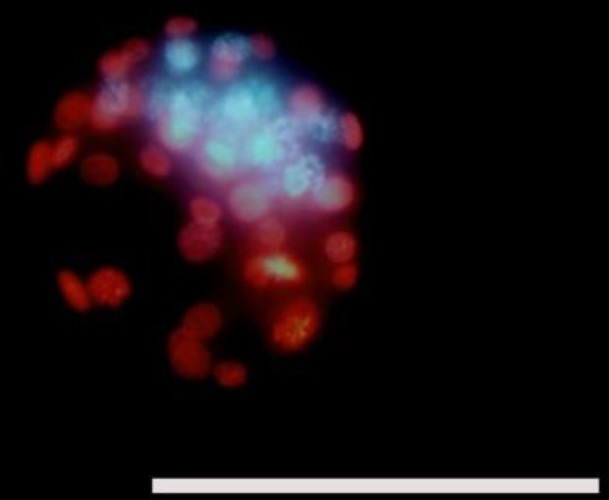
Differential staining in order to determine the number of blastomer cells and inner cell mass cells (ICM), differential staining was performed. The outer trophectoderm (TE) cells were identified by the pink fluorescence of propidium iodide, whereas the ICM cells were recognized by the blue fluorescence of bisbenzimide (Scale Bar=200µm).

As shown in figure 2, in order to determine the number of blastomer cells and ICM cells, differential staining was performed. After observing them with a fluorescent microscope, the number of blastomer cells and inner cell mass cells were counted. All three doses of Kerack decreased the blastomeres quantity in addicted NMRI female mice significantly compared to the control and vehicle groups (p≤0.001). However, in the vehicle group, the number of blastomer cells was less than the control group, while this decrease was not significant (Table II).

**Figure 3:**
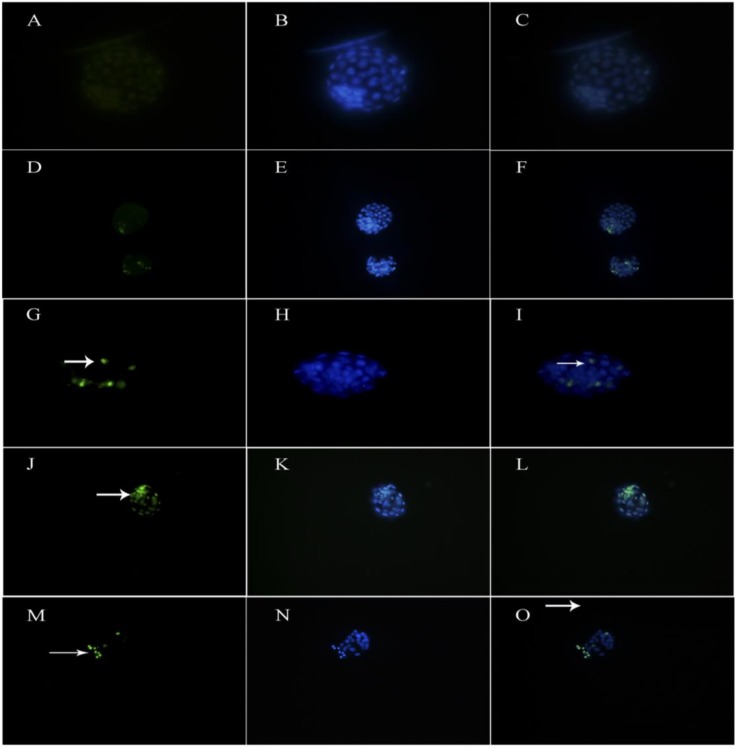
TUNEL assay to detect apoptotic cells. The green cells indicate the TUNEL-positive apoptotic cells. **(A-C)** Negative control embryos (A TUNEL assay; B, DAPI staining; C, merged, ×200). **(D-F) **Positive control embryos (D, TUNEL assay; E, DAPI staining; F, merged×100). **(G-I)** Experimental group I(5mg/kg) (G, TUNEL assay; H, DAPI staining; I, merged×200**). (J-L) **Experimental group II (35mg/kg) (J, TUNEL assay; K, DAPI staining; L, merged×100). **(M-O) **Experimental group III (5mg/kg) (M, TUNEL assay; N, DAPI staining; O, merged×100). Kerack dose-dependently increased meaningfully the apoptotic cells in mouse blastocysts of the experimental group versus the control.

It was shown that increasing the dose of Kerack has no significant effect on the blastomere cells. With regard to the number of the inner cell mass cells, the same results were observed among the groups. All three doses in experimental group have significantly lower inner cell mass cells compared with both control and vehicle groups (p≤0.001) and there was no significant relation between drug dose and the number of inner cell mass cells. Also, in order to determine the dead cells and the DNA fragmentation upon the apoptosis in blastocysts, TUNEL coloring with PI for separation of necrotic cells from apoptotic ones was performed (Figure 3). Figure 3 shows a sample of TUNEL coloring in various groups. 

TUNEL and PI staining showed that Kerack dose-dependently increased meaningfully the apoptotic cells in mouse blastocysts of the experimental group in comparison with the control and vehicle ones (p≤0.001). It was shown that there was no significant difference between 35 mg/kg and others doses (70 mg/kg and 5mg/kg; p=0586); however, there was a meaningful difference between 5mg/kg and 70 mg/kg doses (p=0.12) (Table II).

**Table I:** Development of mouse morulae from addicted and non-addicted mothers in T6 media following 96-h cultivation

**Groups**				**Control**	**Vehicle**	**Exp. I (5 Mg/kg)**	**Exp. II (35 mg/kg)**	**Exp. III (70 mg/kg)**
No.				88	56	53	51	67
24 hr								
	M			3 (3.41)	20 (35.71)	25 (47.17)	7 (13.73)	31 (46.27)
	Eb+Lb			67 (76.14)	35 (62.50)	18 (33.96)	37 (72.55)	34 (50.75)
	Hgb+Hdb			17 (19.32)	0 (0.00)	0 (0.00)	0 (0.00)	0 (0.00)
	D			1 (1.13)	1 (1.79)	10 (18.87)	7 13.73)	2 (2.99)
48 hr								
		M		0 (0.00)	0 (0.00)	1 (1.89)	0 (0.00)	0 (0.00)
		Eb+Lb		28 (31.82)	22 (39.29)	22 (41.51)	30 (58.82)	30 (44.78)
		Hgb+Hdb		53 (60.23)	30 (53.57)	19 (35.85)	14 (27.45)	24 (35.82)
		D		7 (7.95)	4 (7.14)	11 (20.75)	7 13.73)	13 (19.40)
72 hr								
			M	0 (0.00)	0 (0.00)	0 (0.00)	0 (0.00)	0 (0.00)
			Eb+Lb	16 (18.18)	7 (12.50)	5 (9.43)	13 (25.49)	8 (11.94)
			Hgb+Hdb	58 (65.91)	39 (69.64)	37 (69.81)	28 (54.90)	41 (61.19)
			D	14 (15.91)	10 (17.86)	11 (20.75)	10 (19.61)	18 (26.87)
96 hr								
		M		0 (0.00)	0 (0.00)	0 (0.00)	0 (0.00)	0 (0.00)
		Eb+Lb		12 (13.64)	7 (12.50)	1 (1.89)	3 (5.88)	4 (5.97)
		Hgb+Hdb		62 (70.45)	39 (69.64)	38 (71.70)	32 (62.75)	42 (62.69)
		D		14 (15.91)	10 (17.86)	14 (26.42)	16 31.37)	21 (31.34)

Data presented as n (%).

Control, embryos from non-addicted mothers in T6 medium

Vehicle, received only normal saline and lemon juice;

Exp. I, experimental group I received Kerack at a dose of 5 mg/kg after addiction to Kerack;

Exp. II, experimental group II received Kerack at a dose of 35 mg/kg after addiction to Kerack and

Exp. III, Experimental group III received Kerack at a dose of 70 mg/kg after addiction to Kerack.

M: morula

Eb: early blastocyst

Lb: late blastocyst

Hgb: hatching blastocyst

Hgd: hatched blastocyst

D: degenerated embryo.

**Table II:** The effect of Kerack on blastomer, ICM and apoptotic cell number in the blastocyst stage

**Groups **	**Total Cell**	**ICM**	**Apoptotic cell**
Control	65.08±8.42	26.15±5.34	1.46±1.45
Vehicle	56.29±6.16	23.29±3.15	0.29±0.49
Experimental I (5 mg/kg)	46.17±4.15^a^^b^	18.17±3.07 ^a^ ^b^	2.92±2.71 ^a^^b^
Experimental II (35 mg/kg)	46.44±6.46 ^a^^b^	18.11±2.32^a^	5.11±4.86^a^^c^
Experimental III (70 mg/kg)	40.92±7.09^a^	17.17±3.21 ^a^^b^	7.17±2.89 ^a^^b^ ^d^

Control, embryos from non-addicted mothers in T6 medium

Vehicle, received only normal saline and lemon juice;

Exp. I, experimental group I received Kerack at a dose of 5 mg/kg after addiction to Kerack;

Exp. II, experimental group II received Kerack at a dose of 35 mg/kg after addiction to Kerack and

Exp. III, Experimental group III received Kerack at a dose of 70 mg/kg after addiction to Kerack.

ICM: Inner Cell Mass

a Significant difference versus control and vehicle groups (p<0.001).

b Significant difference versus vehicle groups (p<0.05).

c Significant difference versus vehicle groups (p<0.01).

d Significant difference versus exp. 1 (5mg/kg) (p<0.01).

## Discussion

Proper development of the embryo is known to be a pre-requisite for a successful pregnancy. In the present study, the Kerack effects on embryo quality in adult addicted-mice was investigated. Our findings indicated that the Kerack addiction during pregnancy can induce apoptosis, retard preimplantation embryo development, and decrease blastomer cell number at different stages of development. Kerack was purchased randomly from the street and analyzed carefully in the laboratory. Then it was used for laboratory research. In the present study, components of Kerack were caffeine, acetaminophen, morphine, acetyl codeine, codeine, heroin, tebaine, and a small amount of papaverine that was similar to the Kerack components in the study of Farhoudian and coworkers (11).

Similar to our previous study, naloxone was administrated after 7 days of treatment with Kerack in order to confirm the mouse addicted model. Withdrawal signs appeared following administration of naloxone. As a conclusion, the results may be related to the opioid activity of Kerack which is subject to be confirmed in a further study. Other researchers showed that administration of naloxone can reverse morphine effects on decreasing fertility in male rats (20, 21).

There is no standard information about the ingredients of Kerack. As a result of its illegal production, each laboratory has unique formula so a number of components are different and there are few studies in this field. The composition of 18 different samples of Kerack which were purchased from different regions of Tehran province was investigated by Farhoudian and coworkers in 2014. The results showed that Kerack included morphine, codeine, acetyl codeine, heroin, tebaine, acetaminophen, and caffeine (11, 12). These compounds, especially acetyl codeine, have severe effects on the body. For example, hyper use of acetyl codeine can release large amounts of histamine in blood and this can cause anaphylactic shock, paroxysm or convulsions, and death (22). Besides, acetaminophen use in high doses can cause liver necrosis, decreased the testicular size, and disruption of spermatogenesis (22, 23).

So far, different methods have been used to assess embryo quality; to name some, embryo's morphology, natural division, the number of blastomers, as well as chromosomal conditions and blastomers’ nucleus (apoptosis) (2, 24-26). In this study, preimplantation development, the number of blastomers, and a number of apoptotic cells were examined to determine embryo quality in mother rats which were addicted to Kerack. Our findings showed that Kerack decreased embryo quality and postponed embryo development in the preimplantation stage. Moreover, our findings have shown that the number of apoptotic blastomers increased in the addicted group. 

In line with our study, it has been shown that MA abuse during pregnancy increases apoptotic cells (2). Based on previous studies, apoptosis is a natural event in embryonic blastocyst cells; however, its rate is related to embryo quality in blastocyst stage. It means, if the rate of blastocyst cell death is 10% on the 6^th^ day, the embryo has a good quality and if it goes up to 27%, this quality is considered as weak. Some studies have shown that opioid consumption stimulates oxidative stress and will increase reactive oxygen species (ROS) production in different tissues (27-29). It is highly possible that giving Kerack to mice will increase ROS production in ovarian tissue. Studies have indicated that the presence of excessive free radicals in testis can increase the germ cell apoptosis (30-32). 

Malon-di-aldehyde is one of the most important lipid peroxidation products formed by volatile compounds with DNA and proteins and can inhibit the biosynthesis of proteins (33). ROS, such as oxygen, hydrogen peroxide, superoxide anion, and hydroxyl radicals, are the main reasons of cell damages. They have a very important role in oxidative damages. On the other hand, ROS plays an important role at the start of differentiation and cell function by mutations in the mitochondrial genome where is the energy source of cells. 

The role of the opioid system in the process of reproduction can be better known when etiology of infertility and the role of opioids abuse in the fertility are discussed. Endogenous opioids are molecules involved in communication between cells in various organs and tissues of male and female reproductive system. Several studies have proved their regulatory role at some stage of reproduction. In fact, the opioid system plays roles in different levels of reproductive regulation including; 1) Central nervous system level, 2) Testis or ovary level, and 3) Sperm or follicle level. 

To activate, endogenous opioid peptides bind to opioid receptors that are seven transmembranes, G protein-coupled receptors [delta opioid receptor, Mu opioid receptor and kappa opioid receptor]. All of them are involved in the regulation of the pituitary gonadotrophic cell activation and consequently in the regulation of the follicle stimulating hormone and luteinizing hormone release (LH) (34). Follicle stimulating hormone and LH influence directly on the development of follicles and ovulation. Kerack, similar to other opioids, probably reduce LH and estradiol and so they affect the development of the follicle.

In addition, it has been shown that preimplantation embryo is a target of opioid signaling and excessive opioid exposure can disturb normal early embryo development via inhibiting Ca^2+^ influx because the intracellular Ca^2+^ signal is critical for preimplantation embryo development (35).

## Conclusion

We demonstrated that illicit Kerack use in all doses has a harmful effect on embryo quality as well as blastocyst formation, can cause increased apoptotic cells, and also retards preimplantation embryo development. Therefore, Kerack abuse by women in childbearing age results in increased risk of perinatal complications. 
